# Case Report: Use of telitacicept for ocular myasthenia gravis

**DOI:** 10.3389/fimmu.2026.1774173

**Published:** 2026-05-28

**Authors:** Ali Yang, Jing Zhao, Fangfang Li, Rui Pang, Weizhou Zang

**Affiliations:** 1 Department of Neurology, Henan Provincial People’s Hospital, Zhengzhou, China; 2 Department of Neurology, Zhengzhou University People’s Hospital, Zhengzhou, China; 3 Department of Neurology, Henan University People’s Hospital, Zhengzhou, China

**Keywords:** AChR-antibody, case report, ocular myasthenia gravis, telitacicept, treatment

## Abstract

**Background:**

Despite existing treatments of ocular myasthenia gravis (OMG), there remains a need for more effective therapies with fewer side effects. Telitacicept, a novel agent that simultaneously inhibits B lymphocyte stimulator (BLyS) and a proliferation-inducing ligand (APRIL), represents a promising therapeutic candidate for OMG.

**Case presentation:**

In this study, telitacicept monotherapy or combination therapy was administered to three patients with OMG who had shown an inadequate response to, or were intolerant of, standard treatments. All patients demonstrated clinically significant improvement post-treatment. Specifically, both the Myasthenia Gravis Foundation of America-Quantitative Myasthenia Gravis (MGFA-QMG) and the Myasthenia Gravis Activities of Daily Living (MG-ADL) scores showed a marked reduction, indicating decreased disease severity and improved quality of life. Immunological assessments revealed an initial decline in CD19^+^ B lymphocyte counts and acetylcholine receptor (AChR) antibody levels. Interestingly, despite well-controlled clinical symptoms, AChR antibody levels subsequently rebounded and stabilized. Furthermore, pulmonary infections in one patient were not established as causally related to telitacicept.

**Conclusion:**

Telitacicept monotherapy or combination treatment in OMG was associated with favorable clinical improvement and a mild adverse event profile, and its use can be tailored to the individual patient. However, larger-scale and longer-term studies are necessary to confirm these findings and fully establish the role of telitacicept in OMG management.

## Introduction

Myasthenia gravis (MG) is a chronic autoimmune disorder characterized by antibody-mediated attacks on the neuromuscular junction. These attacks directly or indirectly impair acetylcholine receptor (AChR) function, resulting in fluctuating skeletal muscle weakness and fatigue, particularly in the extraocular, bulbar, and limb muscles (1). Conventional treatment strategies include acetylcholinesterase inhibitors, thymectomy, and immunosuppressive therapies.

Ocular manifestations are common in myasthenia gravis, occurring in 15-50% of cases. The most frequent symptoms include fluctuating ptosis, diplopia, and orbicularis weakness. The reported outcomes in adults vary widely, with the rate of ocular myasthenia gravis (OMG) progressing to secondary generalized myasthenia gravis (SGMG) ranging from 11% to 85%, often within the first two years after the onset of ocular symptoms (2). Once the condition progresses to generalized myasthenia gravis (GMG), patients face greater risks and challenges in disease management. Therefore, a primary concern for both patients and clinicians in managing OMG is the risk of conversion to the generalized form. However, while considerable attention is given to GMG, early immunotherapy for OMG is often overlooked by both clinicians and drug developers.

Recent studies have highlighted the critical role of B cells as modulators in MG, given their influence on both antibody production and immune cell interactions (3, 4). Telitacicept, a novel recombinant fusion protein, targets this pathway by combining the ligand-binding domain of the transmembrane activator and CAML interactor (TACI) receptor with the Fc portion of human IgG. It functions as a competitive inhibitor, neutralizing the activities of B lymphocyte stimulator (BLyS) and a proliferation-inducing ligand (APRIL) (5).

While telitacicept is approved in China for GMG, published evidence regarding its efficacy for OMG remains limited. Herein, we document our experience with telitacicept in three cases of OMG, offering both practical insights and potential alternative treatment strategies.

## Case presentation

Patient 1 was a 71-year-old man diagnosed with OMG in April 2020. The diagnosis was based on fluctuating ptosis, positive serology for acetylcholine receptor antibodies (AChR-Ab) (16.3 nmol/L; cut-off 0.4 nmol/L, measured by radioimmunoassay [RIA]) and Titin antibodies (Titin-Ab) (32.17 RU/mL; cut-off 20 RU/mL, also measured by RIA), decremental response on low-frequency repetitive nerve stimulation, and a positive neostigmine test. His initial symptom was right eyelid ptosis, which was more pronounced in the evening and improved with rest. This ocular manifestation led to a diagnosis of OMG, for which he began treatment with pyridostigmine at 60 mg three times daily. His symptoms improved rapidly, prompting him to self-discontinue the medication independently after three months. Approximately three years after the initial diagnosis, his condition evolved with the onset of bilateral ptosis and diplopia. The symptoms persisted despite re-initiating pyridostigmine, necessitating the addition of oral prednisone. While this regimen resulted in several months of improvement and stability, the bilateral eyelid ptosis worsened again when the prednisone dose was tapered to 25 mg daily. This exacerbation led to his admission to our hospital for further assessment and management of his OMG. He was admitted to our department in April 2024.

Upon admission, assessments revealed a Myasthenia Gravis Foundation of America-Quantitative Myasthenia Gravis (MGFA-QMG) score of 7 and a Myasthenia Gravis Activities of Daily Living (MG-ADL) score of 6, indicating a substantial impact on his quality of life and daily functioning. Given the significant clinical activity of his disease, treatment with efgartigimod was initiated. However, after two cycles of efgartigimod combined with oral prednisone, the patient’s QMG score had only decreased from 7 to 4. Due to this suboptimal response, the therapy was switched to telitacicept at a dose of 240 mg weekly. Marked clinical improvement was observed within three weeks. By the five-month follow-up, the prednisone dose had been reduced to 7.5 mg per day, pyridostigmine had been discontinued, and both the QMG and ADL scores had stabilized at 1. No infections or other adverse events were observed.

However, a month later, outdoor activities triggered a pulmonary infection, followed by aggravated ptosis and chest tightness. Arterial blood gas analysis revealed mild hypoxemia, and the QMG score was 4, with one point contributed by respiratory muscle involvement. Symptoms resolved with anti-infectives and temporary pyridostigmine. Two months later, a second pulmonary infection occurred, provoking worsened ptosis and new dysphagia. Despite normal blood gas, the QMG score rose to 7. Treatment with anti-infectives, eculizumab (900 mg), and neostigmine successfully lowered the QMG score to 1. Since then, the patient has been maintained on a regimen of monthly telitacicept (160 mg) and monthly eculizumab (900 mg), with the two agents administered two weeks apart from each other. Both the QMG and ADL scores have remained stable at 1.

Patient 2 was a 52-year-old male diagnosed with OMG in September 2023, based also on fluctuating ptosis, positive AChR-Ab serology (12.1 nmol/L; cut-off 0.4 nmol/L, measured by RIA), a decremental response on low-frequency repetitive nerve stimulation, and a positive neostigmine test. He had been prescribed pyridostigmine bromide, prednisone, and tacrolimus by physicians at an external institution, with doses gradually escalated to 720 mg/day, 60 mg/day, and 6 mg/day, respectively. Despite this aggressive regimen, his symptoms failed to show significant improvement, and his QMG and ADL scores consistently fluctuated around 5. Consequently, four months after disease onset, he received three injections of efgartigimod, which reduced his QMG score to 1. The patient subsequently underwent thymectomy and received two additional postoperative efgartigimod injections.

Nevertheless, his ptosis worsened one month later. Both the QMG score and ADL score have returned to 3 points, prompting him to seek care in our department. He was admitted to our hospital in April 2024. Following the initiation of weekly telitacicept (240 mg), tacrolimus and pyridostigmine bromide were discontinued within one month, and the QMG score stabilized at 1. After four months, the telitacicept interval was extended to every 10 days (maintaining the 240 mg dose), and the prednisone dose was reduced to 7.5 mg/day. Over the following months, the dose of telitacicept was gradually reduced to 160 mg every 10 days and corticosteroids were discontinued, while the patient’s QMG and ADL scores remained stable at 1.

Patient 3 was a 53-year-old woman who first sought formal medical consultation in February 2020, although by her own account, the actual duration of illness may have been longer. Although the results of low-frequency repetitive nerve stimulation were negative, her diagnosis of OMG was based on fluctuating ptosis and diplopia, positive antibody tests (AChR-Ab, titer 1:10, determined by cell-based assay [CBA]), and a positive neostigmine test. However, she promptly self-discontinued pyridostigmine bromide due to adverse effects of nausea and diarrhea and declined corticosteroids or immunosuppressants. Consequently, she continued taking traditional Chinese herbal decoctions for the next four years, during which her ADL score was generally maintained at 2 or 3.

She presented to our department due to worsening left eyelid ptosis and diplopia in June 2024, at which time her QMG and ADL scores were 4 and 5, respectively. Weekly telitacicept at 240 mg was initiated. Due to diarrhea, the regimen was adjusted to 160 mg every 10 days after one month. One month later, her symptoms were well-controlled, and the QMG score had decreased to 1. Subsequently, the telitacicept interval was extended to 160 mg every 2 weeks. After 8 months of treatment, the patient voluntarily ceased medication and has not resumed any since, with both QMG and ADL scores remaining stable.

**Figure 1:**
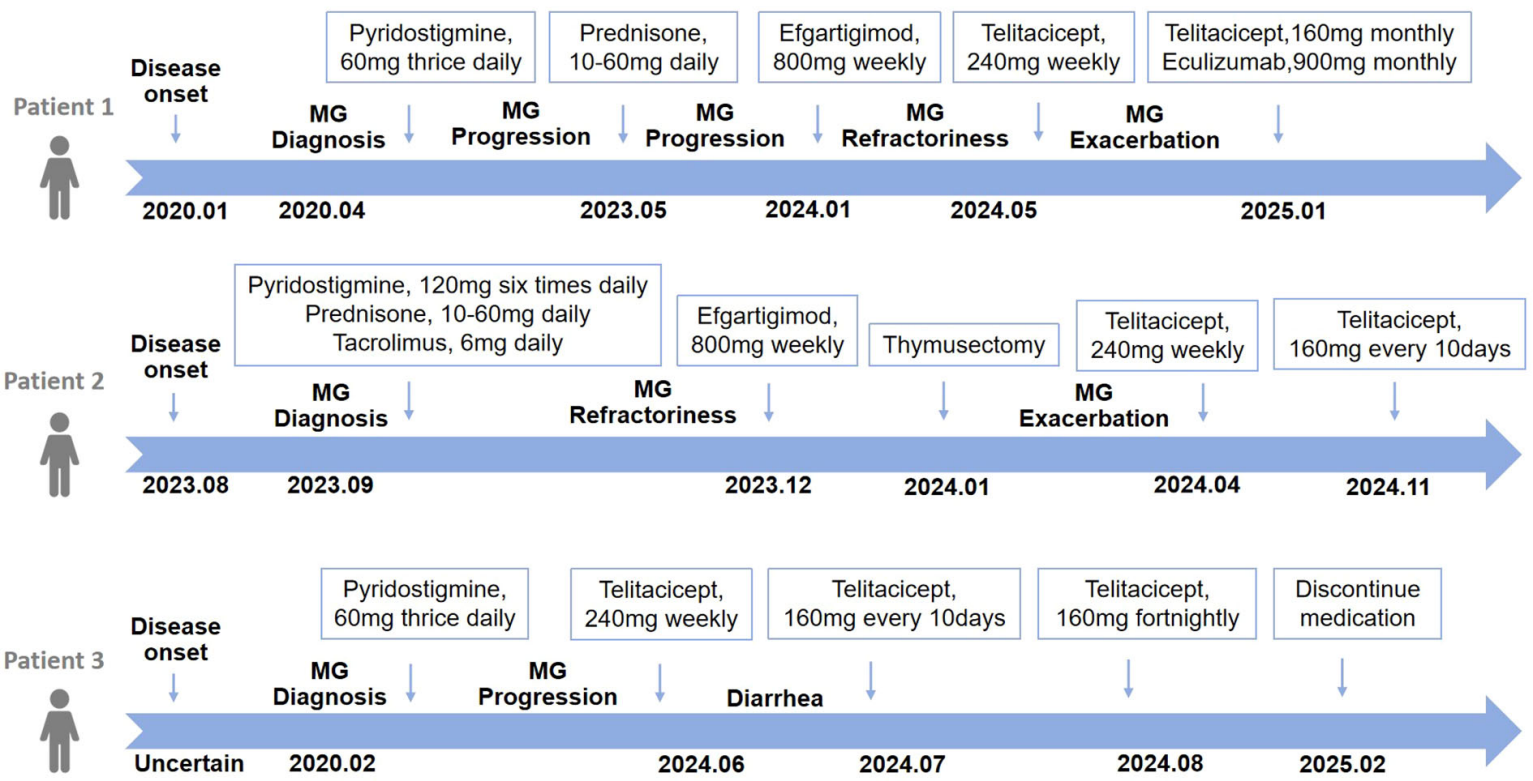
Meducal history of the three OMG patients. OMG, ocular generalized myasthenia gravis.

**Figure 2:**
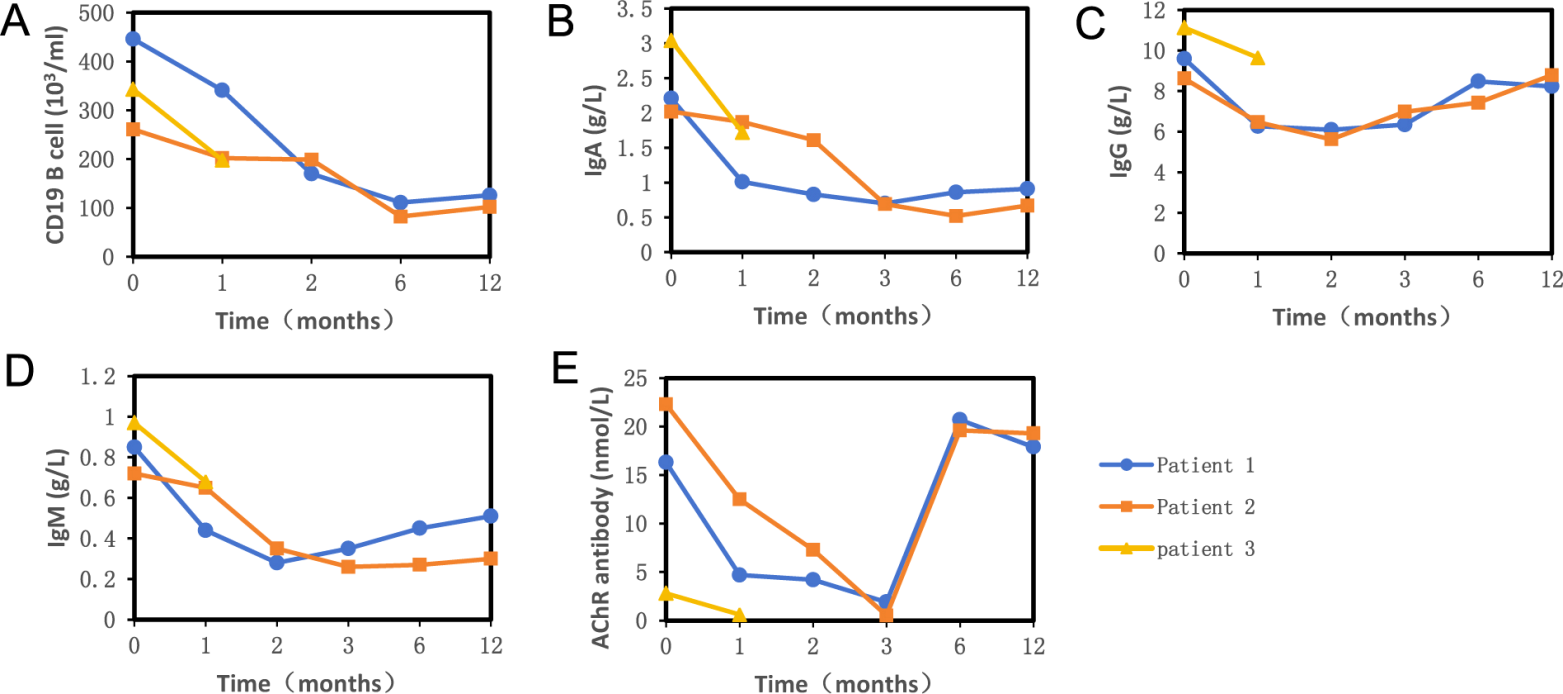
Changes in CD19^+^ B cells and antibody levels. **(A)** Changes in CD19^+^ B cells counts. **(B)** Changes in IgA levels. **(C)** Changes in IgG levels. **(D)** Changes in IgM levels. **(E)** Changes in AChR levels. AChR, acetylcholine receptor; IgA, immunoglobulin A; IgG, immunoglobulin G; IgM, immunoglobulin M.

The clinical characteristics and medical histories of the three patients are summarized in **Table 1** and **Figure 1**. As part of our routine clinical protocol, we dynamically monitored key immunological parameters following the initiation of telitacicept therapy, including CD19+ B lymphocyte counts and the levels of IgA, IgM, IgG, and AChR antibodies. Unfortunately, the third patient was unable to complete the follow-up examinations regularly at our hospital due to residing long-term in another region. Flow cytometric analysis demonstrated a gradual decline in CD19^+^ B lymphocyte counts after telitacicept initiation, followed by stabilization at a lower level. Corresponding reductions in the serum concentrations of IgA, IgM, and IgG were observed in all patients, consistent with the anticipated immunomodulatory effect of telitacicept (**Figure 2**). Notably, two male patients exhibited a reduction in acetylcholine receptor antibody levels to relatively low values within the first six months. However, on reassessment at one year, these levels had rebounded to levels approximating the initial baseline, despite the patients’ clinical status remaining stable at that time.

**Table 1:** Patient characteristics.

Patient no.	Sex	Age of onset	Antibody	Disease duration	Thymoma type	Immunosupressant before telitacicept	Dose of telitacicept	Baseline MGFA-QMG	Baseline MG-ADL
1	M	67	AchR Titin	69 months	None	Prednisone, efgartigimod	240 mg/week→ 160 mg/month (+eculizumab 900 mg/month)	7	6
2	M	52	AchR	25 months	None	Thymectomy, prednisone, tacrolimus, efgartigimod	240 mg/week→240 mg/10 days→160 mg/10 days	5	5
3	F	Uncertain	AchR	Uncertain	None	None	240 mg/week→ 160 mg/10 days→160 mg/2week	4	5

M, male; F, female; AChR, acetylcholine receptor; MGFA-QMG, Myasthenia Gravis Foundation of America Quantitative Myasthenia Gravis Score; MG-ADL, MG-associated Activities of Daily Living score.

## Discussion

This case series evaluates the clinical efficacy of telitacicept, a novel therapy, in three patients with OMG. Although presenting with distinct clinical profiles, all three patients showed significant symptomatic improvement within approximately one month of telitacicept initiation, as evidenced by marked reductions in both MGFA-QMG and MG-ADL scores. Our findings indicate that telitacicept can effectively reduce disease severity and improve daily function, making it a viable treatment option for ocular myasthenia gravis. However, as with other biologic targeted agents, monitoring of B-cell and immunoglobulin levels, as well as clinical adverse reactions, is necessary during treatment. These findings are consistent with a recent case series by Lin et al, who reported that seven patients with refractory ocular myasthenia gravis responded favorably to telitacicept treatment (6).

OMG is an autoimmune disorder of the neuromuscular junction that causes diplopia and ptosis. SGMG develops when patients initially presenting with ocular symptoms progress to weakness in the bulbar, limb, or respiratory muscles. Reported rates of progression from OMG to generalized MG vary substantially across studies (2). This variability may partly explain why some patients with OMG receive only pyridostigmine or a combination of pyridostigmine and corticosteroids. For these patients, particularly those at lower risk of generalization, clinicians must carefully weigh the benefits of immunotherapy against its potential adverse effects. However, once OMG progresses to GMG, disease management becomes more complex and often demands greater medical resources. Despite evidence suggesting that early immunotherapy can reduce generalization risk, other studies contradict this view (7–9). Therefore, the role of early immunotherapy in OMG continues to be debated. Our case series contributes to this debate by demonstrating the potential efficacy of the B-cell-targeting agent telitacicept in this context.

The clinical efficacy of telitacicept monotherapy in Patient 1 was significantly superior to that of efgartigimod, as clinical improvement and successful tapering of prednisone and pyridostigmine occurred before eculizumab was added. As an elderly patient, his disease progressed to generalized MG five years after symptom onset, following two episodes of pulmonary infection. Both infections were preceded by outdoor activities and excessive fatigue. No severe hypogammaglobulinemia was directly linked to the infection episodes. Given that we are unsure of the specific cause of the infection, a tailored regimen of alternating telitacicept and eculizumab was initiated. Over one year of follow-up, this individualized approach has achieved satisfactory disease control without recurrence of pulmonary or other infections. Based on this observation, it is reasonable to infer that the patient’s prior infection was not directly attributable to telitacicept. This case highlights the need for patients with ocular myasthenia gravis for more than two years still require close monitoring. Moreover, the occurrence of adverse events such as infections warrants a comprehensive assessment to determine their relationship to immunotherapy. Patients should also be emphatically advised to avoid potential exacerbating factors, particularly fatigue, even when their condition appears stable. Eculizumab, a humanized monoclonal antibody, inhibits complement-mediated damage by binding with high affinity to terminal complement protein C5. This action blocks the enzymatic cleavage of C5 into C5a and C5b, thereby preventing the proinflammatory effects of C5a and the formation of the membrane attack complex (MAC) initiated by C5b. By mitigating MAC-induced injury, eculizumab may facilitate the restoration of normal anatomical architecture at the neuromuscular junction (NMJ) (10). Notably, in patients with a longer disease course, the potential for structural recovery becomes a critical factor in therapeutic planning. This mechanistic rationale may explain the observed clinical stabilization in our patient following combination therapy with telitacicept and eculizumab, which presumably target distinct pathogenic pathways in a synergistic manner.

Patient 2 presented with persistent ocular symptoms, failed to achieve treatment targets despite multiple therapeutic escalations and concurrent thymectomy. However, within one month of initiating telitacicept, it was possible to taper and discontinue both tacrolimus and pyridostigmine. The patient achieved dual treatment goals within 4 months and has since maintained highly stable disease control. This case highlights the variability in treatment responses can vary significantly depending on the specific immunotherapy agent used. Similar to the generalized form, OMG can also present in a refractory form. Without reliable biomarkers for drug selection, treatment requires a personalized empirical approach. The choice of therapy, including novel biologics, should be guided by the patient’s specific clinical characteristics and therapeutic needs.

Patient 3’s overall ocular symptoms showed no significant fluctuation, and no systemic conversion occurred for a considerable period of time. Additionally, the acetylcholine receptor antibody titer was low. Therefore, after comprehensive evaluation, the patient was determined to be at low risk of disease exacerbation. However, the patient developed significant intolerance to pyridostigmine and declined corticosteroid or conventional immunosuppressive therapy due to personal concerns. Telitacicept was subsequently initiated as monotherapy, resulting in rapid symptomatic improvement. After 8 months of treatment, the medication was discontinued. The patient has since remained clinically stable for 9 months without any therapy, demonstrating a substantial and sustained benefit from the intervention. Nevertheless, regular long-term follow-up remains essential for this patient.

Telitacicept demonstrated a favorable safety profile in our cohort. Although the first patient experienced two episodes of pulmonary infection, no further infectious events occurred during the subsequent year, while his condition was stabilized on the alternating regimen of telitacicept and eculizumab. Immunologically, telitacicept treatment induced a reduction in CD19+ B lymphocyte counts and decreased serum levels of IgA, IgM, IgG, and AChR antibodies. These findings are consistent with the drug’s known mechanism of action, which involves neutralizing the cytokines BLyS and APRIL to inhibit B-cell-mediated humoral immunity and reduce pathogenic autoantibody production. However, the observed decline in IgG levels raises consideration of potential infection risk. Therefore, we recommend pre-treatment vaccination (especially for influenza and pneumococcal pneumonia) and periodic immune function monitoring as essential preventive measures.

It is worth noting that although the first two patients showed a significant initial decline in antibody titers upon treatment, both experienced a rebound to near-pretreatment levels during follow-up, despite achieving eventual clinical stability. This observation underscores the complex and potentially discordant relationship between myasthenia gravis antibody titers, disease severity, and relapse risk. While serum antibody titers are a poor predictor of disease severity in MG (11), evidence suggests they may be associated with disease progression and relapse risk (12). This imperfect correlation is attributed to several factors. Leading theories include, but are not limited to (13): (1) The heterogeneity of AChR subunits and epitopes, distributed across various postsynaptic and ion channel sites, which results in antibodies of differing pathogenic potential. Consequently, the total antibody titer may not reflect the net pathogenic burden. (2) Individual AChR antibodies can engage multiple pathogenic mechanisms simultaneously. These, among other factors, contribute to the dissociation between serological levels and clinical manifestations. A recent study (14) confirmed female patients exhibited higher titers than males, with levels inversely correlated with age-a pattern absent in males, suggesting sex-specific pathophysiology. Furthermore, significant sex-based immunodominance: males predominantly showed α immunodominance, whereas females primarily exhibited γ immunodominance. Specifically, γ immunodominance correlated with elevated titers and increased disease severity, pointing to a distinct clinical and immunological subgroup. These complexities underscore the need to develop a multidimensional composite parameter to serve as a reliable prognostic biomarker.

Interestingly, the two male patients in this study showed a suboptimal response to efgartigimod. While clinical and real-world evidence confirms the overall efficacy of this FcRn antagonist in MG (15), some patients remain poor responders. Potential explanations include: Limited tissue penetration: Efgartigimod effectively clears IgG from the circulation but may not fully access antibodies sequestered in tissue microenvironments (e.g., behind the blood-ocular barrier in OMG), leaving local neuromuscular junctions vulnerable to damage. Unchecked antibody production: The drug accelerates antibody degradation but does not suppress *de novo* antibody synthesis. In patients with active thymic pathology, ongoing antibody production may outpace drug-mediated clearance. Pathogenic mechanisms beyond IgG: In patients with low baseline AChR antibody titers, symptoms may be driven by non-antibody pathways (e.g., T-cell-mediated damage, complement activation)—targets that efgartigimod does not act on (16). These factors, among others, may contribute to treatment resistance in certain MG patients. In contrast, telitacicept targets BLyS and APRIL to inhibit the maturation and differentiation of B cells and plasma cells at multiple stages, thereby suppressing the production of autoantibodies at an upstream level. Additionally, due to the expression of TACI receptors on T cells, telitacicept also inhibits T cell activation (17). These mechanisms likely explain the marked improvement in patient symptoms observed in this study.

## Conclusion

In summary, while the condition of all three patients stabilized eventually, the notable contrast in their disease trajectories raises important considerations regarding underlying mechanisms and management. The management of OMG should be based on a thorough assessment of clinical features and patient needs, leading to an individualized strategy tailored to prevent progression to generalized disease. Telitacicept demonstrated promising efficacy in treating OMG, improving both clinical scores and quality of life while exhibiting a favorable short-term safety profile. Individualized dosing and combination strategies may further optimize outcomes. Our observations support telitacicept as a valuable option for OMG, but larger prospective studies are needed to confirm its role, especially regarding long-term progression to generalized disease.

## Patient perspective

Although all three patients presented primarily with ocular symptoms, their occupational and social responsibilities led to a relatively high demand for effective treatment. When symptoms were inadequately controlled, each experienced varying degrees of anxiety and depression. Following adjustment of the treatment regimen, however, all reported gradual clinical improvement. This symptomatic relief was accompanied by marked improvements in mood and overall outlook. The patients expressed satisfaction with the care provided at our hospital, the thorough explanation of their condition, and the introduction of telitacicept as a therapeutic option. Written informed consent for individualized therapy was obtained from all patients.

## Data Availability

The original contributions presented in the study are included in the article/supplementary material. Further inquiries can be directed to the corresponding authors.
